# Special Issue: Repetitive DNA Sequences

**DOI:** 10.3390/genes10110896

**Published:** 2019-11-06

**Authors:** Sarah E. Lower, Anne-Marie Dion-Côté, Andrew G. Clark, Daniel A. Barbash

**Affiliations:** 1 Department of Biology, Bucknell University, Lewisburg, PA 17837, USA; s.lower@bucknell.edu; 2 Department of Molecular Biology and Genetics, Cornell University, Ithaca, NY 14850, USAac347@cornell.edu (A.G.C.); 3 Biology Department, Université de Moncton, Moncton, NB E1A 3E9, Canada

**Keywords:** repetitive DNA, transposable element, heterochromatin, genome evolution, genomic conflict

## Abstract

Repetitive DNAs are ubiquitous in eukaryotic genomes and, in many species, comprise the bulk of the genome. Repeats include transposable elements that can self-mobilize and disperse around the genome and tandemly-repeated satellite DNAs that increase in copy number due to replication slippage and unequal crossing over. Despite their abundance, repetitive DNAs are often ignored in genomic studies due to technical challenges in identifying, assembling, and quantifying them. New technologies and methods are now allowing unprecedented power to analyze repetitive DNAs across diverse taxa. Repetitive DNAs are of particular interest because they can represent distinct modes of genome evolution. Some repetitive DNAs form essential genome structures, such as telomeres and centromeres, that are required for proper chromosome maintenance and segregation, while others form piRNA clusters that regulate transposable elements; thus, these elements are expected to evolve under purifying selection. In contrast, other repeats evolve selfishly and cause genetic conflicts with their host species that drive adaptive evolution of host defense systems. However, the majority of repeats likely accumulate in eukaryotes in the absence of selection due to mechanisms of transposition and unequal crossing over. However, even these “neutral” repeats may indirectly influence genome evolution as they reach high abundance. In this Special Issue, the contributing authors explore these questions from a range of perspectives.

Repetitive DNAs include both short and long sequences that repeat in tandem or are interspersed throughout the genome, such as transposable elements (TE), ribosomal rRNA genes (rDNA), and satellite DNA. Repetitive DNA is ubiquitous in eukaryotic genomes, but despite this universality, their possible functions and predictable patterns of evolution remain relatively poorly characterized across taxa. Empirical evidence suggests important roles of repetitive DNA in chromosome stability and segregation, as well as gene regulation. Theory predicts roles of both neutral processes (unequal crossing over, gene conversion) and selection, as well as selfish (non-Mendelian) transmission, in determining patterns of sequence variation in repetitive regions. Despite a wealth of theory, until recently, this fraction of the genome has remained largely overlooked due to technological constraints on sequencing and quantifying repetitive DNA genome-wide. With the advent of high-throughput sequencing technologies, this portion of the genome has become more accessible, though inherent biases due to sequencing chemistry and computational identification pipelines remain challenges.

In this special issue, 11 articles review the evolution and function of the different classes of repetitive DNA and empirically investigate their predicted functions and evolutionary patterns from a variety of perspectives. Two articles approach TE evolution from different angles—Blumenstiel [1] describes the life cycle of a TE and uses this analogy to develop predictions for how TEs evolve, using known examples to describe persisting TEs as quickly proliferating genome invaders, long-lasting residents, and even as “resurrectors” from previously “dead” copies. In another review, Bourgeois and Boissinot [2] synthesize perspectives on the roles of adaptive and non-adaptive processes in TE evolution and offer ways forward to model TE evolution at the population level.

Two studies test predictions about TE evolution using a macroevolution approach. Wu and Lu [3] first develop a new pipeline for identifying transposable elements and then apply it to examine TE proliferation and diversification across 500 million years of arthropod evolution. They introduce the Arthropod TE database as a resource for TE consensus sequences for the community to use and build on. Bohman et al. [4] provide a genome assembly for the Blue-capped Cordon-Bleu, a small East African finch, whose karyotype and annotated transposon content enable new detailed examination of TE evolution in birds, particularly relatives of the model zebra finch. Their results highlight the utility of employing a comparative approach to investigate TE evolution. Together, these papers offer a dynamic view of TE evolution.

Three papers examine the role of adaptive and non-adaptive processes in TE evolution using genomic and functional approaches. Taking a computational approach, Pettersson and Jern [5] find a greater role for neutral evolution rather than selection in endogenous retrovirus (ERV) diversification across domestic chicken lineages. In contrast, Radion et al. [6] use functional and genomic analyses to examine the transcriptional regulation of piRNA clusters and TEs and find evidence for selective constraints. Lannes et al. [7] provide evidence for links between TE presence/absence and regulation of their activity via epigenetic modifications, implicating selection on their regulation. Together, these papers demonstrate the interplay of selection and neutral processes in different groups and emphasize the need for more studies to test broadly applicable “rules” for TE evolution.

Four papers focus on the evolution and function of other less-studied repetitive DNA types. Symonová [8] reviews studies of rDNA, from their function to their use in phylogeny and integrates these perspectives to provide a wider view of rDNA importance and evolution. Benetta et al. [9] synthesize recent work on the non-Mendelian transmission of repetitive facultative (B) chromosomes. Miga [10] reviews recent work on the links between satellite DNA and disease, highlighting the importance of their study to human health. Hartley and O’Neill [11] discuss the evolution and function of satellite DNA and TEs in centromeres. These papers highlight overlooked types of repetitive DNA and identify key challenges to move the field forward.

This special issue demonstrates the benefits of applying multiple perspectives to tackle questions about repetitive DNA evolution, function, and adaptation. They paint a picture of the complex processes involved and reveal the need for additional work. With more affordable sequencing, and a growing arsenal of genetic tools and widely-available annotation databases, it is a promising time to tackle fundamental questions about repetitive DNA with important implications for our understanding of the fundamental rules of chromosome segregation, genome evolution, and human health. We would like to thank all of the authors and reviewers for their contributions to this issue.
